# The effect of temperature on language complexity: Evidence from seven million parliamentary speeches

**DOI:** 10.1016/j.isci.2024.110106

**Published:** 2024-06-13

**Authors:** Risto Conte Keivabu, Tobias Widmann

**Affiliations:** 1 Max Planck Institute for Demographic Research (MPIDR), Konrad-Zuse-Straße 1, 18057 Rostock, Germany; 2 Aarhus University, Bartholins Allé 7, 8000 Aarhus C, Denmark

**Keywords:** Earth sciences, Environmental science, Linguistics

## Abstract

Climate change carries important effects on human wellbeing and performance, and increasingly research is documenting the negative impacts of out-of-comfort temperatures on workplace performance. In this study, we investigate the plausibly causal effect of extreme temperatures, i.e., out-of-comfort, on language complexity among politicians, leveraging a fixed effects strategy. We analyze language complexity in over seven million parliamentary speeches across eight countries, connecting them with precise daily meteorological information. We find hot days reduce politicians’ language complexity, but not cold days. Focusing on one country, we explore marginal effects by age and gender, suggesting high temperatures significantly impact older politicians at lower thresholds. The findings propose that political rhetoric is not only driven by political circumstances and strategic concerns but also by physiological responses to external environmental factors. Overall, the study holds important implications on how climate change could affect human cognitive performance and the quality of political discourse.

## Highlights

• Heat has been associated with decreased productivity and cognitive performance
• We find warmer temperatures to reduce language complexity of politicians
• In Germany, we observe a larger effect of warm days on older politicians
• Climate change could reduce the speech complexity and productivity of politicians

## Graphical abstract

**Figure:**
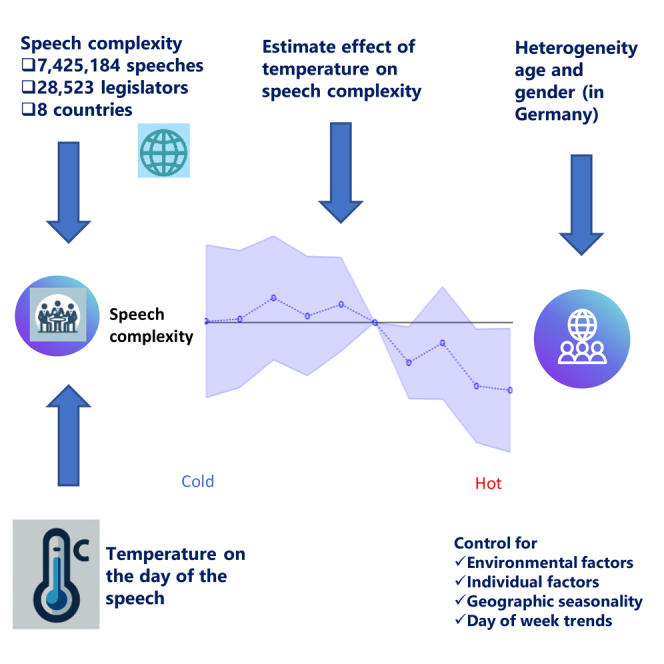

## Introduction

Climate change is causing a steady increase in temperatures with consequences on several dimensions of human wellbeing.^1^^,^^2^ Previous studies showed extreme temperatures (i.e., out of comfort), especially heat, to increase the incidence of negative health outcomes such as morbidity^3^ and mortality.^4^^,^^5^ Additionally, studies exposed extreme temperatures to be deleterious also for a large array of other health related outcomes, affecting expressed sentiment,^6^^,^^7^^,^^8^ externalizing behavior,^9^^,^^10^^,^^11^^,^^12^ cognitive performance of students and office workers,^13^^,^^14^^,^^15^^,^^16^^,^^17^^,^^18^^,^^19^ learning,^20^^,^^21^ time allocation,^22^ absenteeism,^23^^,^^24^ hate speech,^25^ decision making,^26^ and manual labor productivity.^23^^,^^27^ In this article, we leverage information on parliamentary speeches to focus on one of these outcomes: language complexity (which could serve as a potential proxy for cognitive performance).

Speech complexity of political discourse has been studied extensively in social science research. Looking at long term developments, studies indicate that language complexity of political language has steadily decreased over the past 200 years.^28^ Furthermore, concerns are often brought forward in connection to rising populist movements and prominent populist leaders, who allegedly use less complex political language in order to strategically appeal to and manipulate their voters. Studies provided evidence for populists’ use of simpler political language^29^ and for the consequences of less complex language on voters.^30^

Further evidence shows that linguistic habits can be dependent on personality traits^31^^,^^32^ and political ideology^33^ of speakers. A study analyzing parliamentary speeches from different European countries and European prime ministers shows that speakers from culturally liberal parties use more complex language than speakers from culturally conservative parties.^34^ Moreover, studies have shown that factors such as incumbency^35^ or the temporal focus of messages^36^ matter for the type of language used in political discourse. Additionally, a number of studies suggest that certain political styles are used strategically, for instance, to address larger general audiences^37^ or specific social groups,^38^ to refer to their past achievements,^35^ or to distance themselves from political rivals.^39^^,^^40^ Importantly, however, most of these studies emphasize the strategic character of political language or perceive communication styles as determined by intrinsic characteristics of politicians.

Yet, environmental factors, unrelated to strategic considerations, have been hitherto largely neglected but could have a substantial impact on human behavior. One study researched the impact of air pollution on politicians' language complexity in Canada.^41^ The authors find that high levels of particulate matter 2.5 decrease speech complexity, which is used to capture the cognitive performance of politicians. In a similar fashion, hot temperatures have been shown to also affect other important cognitive processes, such as decision-making. Studies from the United States and from India both indicate that judges come to different verdicts under high temperatures,^42^^,^^43^ but we warrant that results for the United States have been shown to not be stable due to coding errors and when comprising a larger dataset.^44^ Interestingly, the controlled climatic environment of courts does not prevent temperature from influencing these decisions. Studies on the impact of extreme temperatures on politicians’ speeches are, to the best of our knowledge, not existing. Yet, based on the research above, we expect high temperatures to be also relevant for politicians’ level of speech complexity.

Prior work has used individual’s speech complexity as a marker of cognitive performance.^41^ The intricate structure and subtlety inherent in language, including sentence and word length, can be perceived as mirroring the cognitive processes behind their creation. As such, the manifestation of shorter sentence or word patterns may be indicative of diminished cognitive functionality. Accordingly, a decline in cognitive performance may result in a corresponding decrease in speech complexity, highlighting the intertwined nature of cognitive and linguistic performance. Nevertheless, we acknowledge that the measure is a proxy of cognitive performance and not a perfect measure of it.

We put forth the hypothesis that extreme temperatures negatively affect speech complexity among politicians, thereby leading to a simplification of political rhetoric. This hypothesis builds upon previous research that has established a connection between temperature and various cognitive tasks,^18^^,^^45^ as well as test scores.^15^^,^^46^ These studies suggest that extreme temperatures result in a significant decrease in cognitive performance. Interestingly, heat appears to have a more pronounced effect than cold on office workers.^47^ However, research has also shown that cold temperatures adversely impact the performance of university students^13^ and older adults.^48^ Given the physiological sensitivity of humans to temperature, we anticipate that politicians' behavior will be influenced not only by strategic considerations but also by their physiological responses. Therefore, we expect that extreme daily temperatures, both cold and hot, will affect political rhetoric in parliament due to their impact on the physiological and psychological state of politicians.

We further expect marginal effects based on age and gender. We expect politicians aged above 65 to exhibit larger effects of extreme temperatures on their language complexity. Extensive epidemiological research has demonstrated that individuals aged 65 and above exhibit a higher vulnerability to both cold and heat.^49^ This increased susceptibility is attributed to their weaker cardiovascular systems, which are less capable of managing temperature-related stress.^50^ Indeed, numerous studies have reported a significant rise in morbidity-related outcomes, such as hospitalization or mortality, among this age group.^51^ Additionally, these studies have observed a decline in cognitive performance when temperatures deviate from comfortable levels.^52^ As a result, age should be considered a stratifying factor when examining the impact of extreme temperatures on politicians' speeches. Specifically, politicians aged 65 and above are likely to be most affected by extreme temperatures.

Further research has shown that women generally prefer slightly warmer temperatures compared to men,^53^ and they are more likely to express dissatisfaction with their thermal comfort in a room.^54^ Additionally, women’s cognitive performance tends to be more negatively affected by cold temperatures.^55^ In contrast, men experience a more significant decrease in performance under hot temperatures. Consequently, we predict that the language complexity of female politicians may be more significantly impacted by extremely cold temperatures. In contrast, we expect the language complexity of male politicians to decrease in response to heat.

## Results

In our analysis, we estimate the plausibly causal effect of temperature on language complexity using ordinary least squared (OLS) regression with a fixed effects (FE) strategy. More precisely, we use individual fixed effects to inquire how variation in the temperature on the day of the speech orthogonally affects politicians’ rhetoric. Also, we use month-by-location FE to capture possible seasonal patterns specific to each location in speech quality and day of week FE to capture fatigue that might accumulate during the week and impact speech complexity and cluster standard errors at the month-by-location level. Similarly, we include individual and meteorological control variables to correct for additional biases that could affect our estimates (we further discuss the estimation strategy and other model specifications in the Methods section). Our data consists of all parliamentary speeches in Washington D.C. (United States, 1950–2017), London (United Kingdom, 1989–2019), Vienna (Austria, 1996–2018), Amsterdam (Netherlands, 1996–2018), Wellington (New Zealand, 1989–2019), Copenhagen (Denmark, 1996–2018), Madrid (Spain, 1996–2018), Bonn (West Germany, 1950–1999), and Berlin (Germany, 1999–2019) comprising 7,425,184 speeches from 28,523 politicians (we provide more details on the data source in the methods section). The analysis of marginal effects, however, is based solely on Germany as the German dataset^56^ is the only one providing information on age and gender. We capture language complexity using the Flesch-Kincaid score^57^ (additional complexity scores are reported in the Supplementary materials). The Flesch-Kincaid score indicates the number of years of education needed to accurately understand a given text. Temperature is included using the average temperature on the day of the speech. We opt for dummy indicators for temperature exposure instead of a continuous measure as we expect a non-linear relationship. We construct dummy variables using temperature bins based on the following 10 temperature ranges: <0°C; 0°C–3°C; 3°C–6°C; 6°C–9°C; 9°C to 12°C; 12°C–18°C (comfort zone); 18°C to 21°C; 21°C to 24°C; 24°C to 27°C; >27°C. The coefficients should be interpreted as the effect of out of comfort temperature (12°C–18°C) on the speech complexity of an individual politician relative to a day in the comfort zone. We provide summary statistics for our main variables in Table S1 and describe the observations by country in Table S2. Additionally, in Figures S1 and S2 we show the pattern over time in the Flesch-Kincaid score and average temperature by cities and in Figure S3 we present the distribution of the temperature ranges in our estimation sample and in Figure S4 for Germany only.

**Figure 1:**
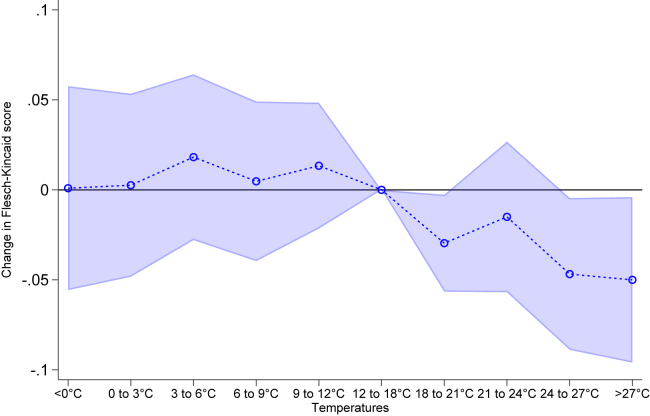
Effect of mean temperature on speech complexity Note: The figure shows the results of exposure to the mean temperature ranges on the Flesch-Kincaid score. We added month-by-location, day of week and politician FE. Moreover, we control for precipitation, relative humidity and wind speed. Standard errors clustered at the month-by-location level. We report 95% confidence intervals.

In Figure 1 we show the relationship between temperature and language complexity of individual politicians, as measured by the Flesch-Kincaid score. We can observe the largest decrease in the language proficiency of politicians at high mean temperatures (24°C–27°C and >27°C) and to a lower extent as well at 18°C–21°C, but not on cold days. In substantive terms, heat (>27°C) decreases the Flesch-Kincaid score by −0.05 or −0.45 percent. This corresponds to approximately a half month of lower educational attainment. Notably, our estimates remain robust to alternative model specifications employing alternative fixed effects or clustering of standard errors (Table S3).

Compared to previous studies researching the effect of environmental factors on language complexity, our analysis reveals more modest effects. For example, employing a model similar to ours, Heyes et al. (2019) also investigated language complexity among politicians. They discovered a 2.3 percent reduction in the Flesch-Kincaid score on days characterized by high PM2.5 levels. This discrepancy may stem from variations in the study contexts, especially since we are considering eight different countries whereas Heyes et al. (2019) focus only on Canada, or from the possibility that air quality has a larger detrimental effect than heat. Interestingly, Heyes et al. (2019) report in the Appendix a positive effect of temperature and temperature squared on the Flesch-Kincaid score, which indicates that politician speeches become more complex with high temperatures. However, our results from eight countries employs a modeling strategy that better captures the non-linear effects of temperature and results contrast these findings, showing that heat negatively impacts language complexity. Although the magnitude of the effect we find is small, it points toward an interesting physiological or psychological response to heat stress that affects linguistic complexity, albeit to a minor extent. Since the Flesch-Kincaid score takes both average sentence length as well as average word length into consideration, we additionally tested both components separately (see SM Figure S5). This exercise indicates that heat influences mostly word length, by 3.3 percentage points, compared to null effects for the sentence length. Thus, politicians use significantly shorter words on hot days (for a more thorough discussion of the suitability of language complexity scores see the methods section). Additionally, we observe the impact of heat on the Flesch-Kincaid score to be concentrated on the day of the speech, as we do not observe temperatures on the previous three days to affect our outcome suggesting an acute effect on the day of the speech (see SM Figure S7).

**Figure 2:**
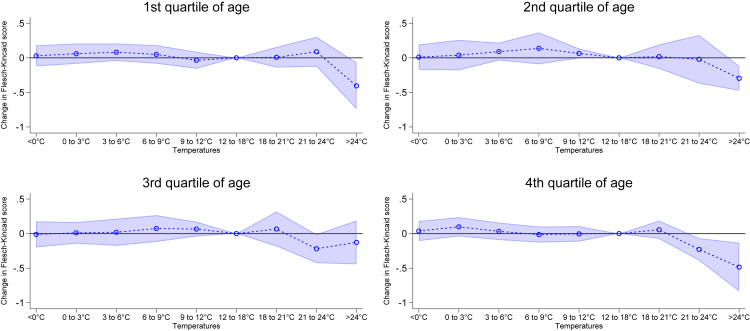
Temperature and language complexity by age in quartiles Germany Note: The figure shows the average marginal effects of the interaction between age categories based on quartiles and exposure to the temperature ranges on the Flesch-Kincaid score in Germany. The first quartile comprises speeches made by politicians aged 19 to 44, the second quartile aged 45 to 50, the third quartile aged 51 to 56, and fourth quartile aged above 57. We added month-by-city, day of week and politician FE. Moreover, we control for precipitation, relative humidity and wind speed. Standard errors clustered at the politician level. We report 95% confidence intervals.

In Figure 2, we expose the stratified effect of temperature on the Flesch-Kincaid score adding an interaction with four age categories grouped based on quartiles of age for political speeches in Germany (We provide summary statistics for the variables for Germany in Table S4). More precisely, the first quartile comprises speeches made by politicians aged 19 to 44, the second quartile aged 45 to 50, the third quartile aged 51 to 56, and fourth quartile aged above 57. Contrary to our expectations, we do not observe a larger effect size of heat (>27°C) for politicians in the fourth quartile in the age distribution (results also reported in SM Table S5). The results highlight heat to affect all politicians without any major differences by age. Nevertheless, we observe the impact of temperature between 21°C and 24°C only on older politicians suggesting that high temperatures begin to have significant impacts on their speech complexity at a lower threshold.

**Figure 3:**
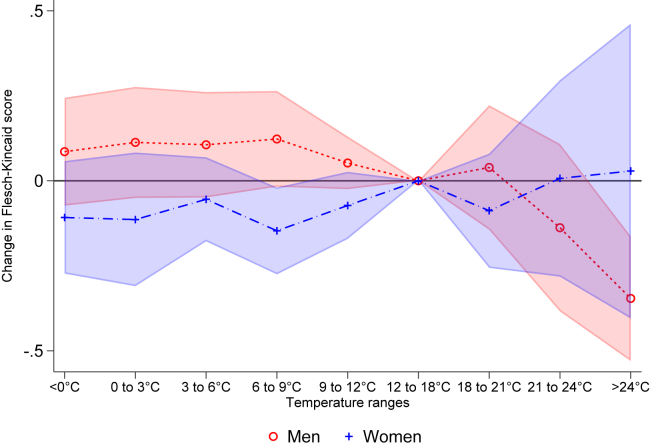
Temperature and language complexity by gender in Germany Note: The figure shows the average marginal effects of the interaction between gender categories and exposure to the temperature ranges on the Flesch-Kincaid score in Germany. We added month, day of week and politician FE. Moreover, we control for precipitation, relative humidity and wind speed. Standard errors clustered at the politician level. We report 95% confidence intervals.

Lastly, also relying on German data, we examine the marginal effects of gender as studies have shown that women are more detrimentally affected by cold exposure and men are more affected by heat exposure.^55^ In Figure 3, we report results of the marginal effects of temperature on the Flesch-Kincaid score adding an interaction with gender. We observe a larger effect size of hot temperatures (>24°C) for male politicians and smaller for female politicians (results also reported in SM Table S6). Nevertheless, such differences are not statistically different at the 95% level, possibly due to the underrepresentation of women in parliaments leading to large confidence intervals for such exposure. Also, we do not observe cold temperatures to decrease language complexity more for women compared to men. Overall, these results are suggestive of the experimental evidence on the higher susceptibility of men to heat provided by previous studies.^55^

## Discussion

In this study, we examined the effect of extreme temperatures on the language complexity of politicians by analyzing over 7 million parliamentary speeches from more than 28,000 politicians across 8 countries. The findings reveal that hot temperatures adversely impact speech complexity, resulting in less complex political speeches. Furthermore, we explored how the impact of temperature on language complexity varies based on age and gender. Our data for Germany, indicates that the detrimental effect of heat is ubiquitous across the age distribution, but older politicians (4th quartile of the age distribution) are more sensitive to warm days (21–24°C) suggesting a lower threshold of vulnerability to high temperatures. Also, we found a larger effect size of heat (>24°C) for male politicians, corroborating previous experimental studies,^55^ but these results are not statistically different at the 95% level from female politicians. Our findings thus provide initial evidence that the complexity of political speech - a crucial element of political discourse - are influenced not only by strategic political and legislative considerations, but also by physiological processes triggered by external environmental factors.

The exact mechanisms through which these effects occur are beyond the scope of our paper. However, multiple pathways can be discussed. In general, extreme temperatures represent a higher burden on the thermoregulatory system that has to work more to keep the body temperature at about 37°C.^58^ But how can extreme *outdoor* temperatures have an effect on political speeches held *indoors*? As suggested by Heyes and Saberian (2019), extreme outdoor temperatures can influence behavior by short exposures during outdoor breaks or during the commute. Another explanation could be that extreme outdoor temperatures could alter behavior and cause politicians to stay inside buildings, which can lead to a lack of fresh air which has been linked to reduced cognitive function.^59^ Similarly, extreme temperatures can also disrupt sleep,^60^^,^^61^ particularly for the elderly,^62^ with important consequences on cognitive performance.^63^

### Limitations of the study

Here, we share some common limitations in the literature but offer possible ideas to fill these gaps in future research. First, we were not able to test specific mechanisms explaining the impact of temperature on speech complexity. Investigating the exact pathways of how outside temperatures can influence political behavior indoors represents a fruitful avenue for future research. In addition, future research could also focus on other political outcomes besides speech characteristics, such as the emotionalization of political speech or legislative decision-making, which can carry far-reaching consequences for political discourse and societies.

Another limitation of our study concerns the process of speech preparation and delivery. Many political speeches are crafted well in advance of their presentation, often with substantial input from advisors and assistants. This advanced preparation raises questions about the direct impact of environmental conditions, such as high temperatures on the day of the speech, on the complexity of the delivered speech. A possible explanation could be that the physiological and psychological effects of high temperatures influence a speaker to simplify speech or diverge from prepared remarks due to impaired cognitive function and comfort. Future research could explore this area further by comparing the complexity of prepared texts with that of delivered speeches under varying temperature conditions, thereby providing a more nuanced understanding of how environmental factors influence speech complexity.

Furthermore, another limitation of our analysis involves the use of the Flesch-Kincaid readability index (and related scores) to evaluate the complexity of spoken political speeches. Originally designed for assessing written text readability, the Flesch-Kincaid index may not capture all dimensions of spoken language complexity, such as intonation, pacing, and non-verbal cues, which play significant roles in speech comprehension and engagement. We acknowledge that this approach does not fully encapsulate the nuances of oral discourse. Consequently, we recommend that future research explore the development and application of more sophisticated linguistic and computational models that are specifically designed for analyzing spoken language by looking at paralinguistic elements.

Lastly, we face some limitation related to the data used. Considering the data on politicians, due to constraints in accessing consistent and reliable demographic data across various countries, our analysis of marginal effects by age and gender was limited to Germany. Future research could overcome this limitation by leveraging collaborations or emerging digital archives to incorporate comprehensive demographic data from multiple countries, enriching the understanding of environmental impacts on speech complexity in diverse contexts. Also, the data are not available for the same period for all countries, as for the United States and Germany data are available from 1950, but for the other countries this is available mostly from the 1980s or 1990s. Arguably, there could be differences in the impact of temperature over time, due to adaptation or stronger effects of heat in later decades. Furthermore, the structure of the language could have changed, becoming less complex over time. We run interaction analysis between the temperature exposure with years grouped in the categories 1950–1967; 1968-1985; 1986–2003; 2004-2019 and we did so for the full sample and separately for the United States and Germany, i.e., the countries for which we have data for the whole period. As shown in SM Table S7, we observe a positive interaction coefficient for the later periods compared to the years 1950–1967. In particular, such results are more substantive and robust for the analysis for the United States for which we observe no effect of days above >27°C in the period 2004–2019. In line with previous studies on temperature and mortality, the results suggest a weakening of the impact of heat on political speeches in the later period.^64^ Related to the data on environmental factors, we control for wind speed, relative humidity and precipitation, but other exposures such as air pollution (e.g., ozone, PM2.5), cloud cover or day light could correlate with high temperatures and bias our estimates. However, we tested how results change when controlling for air pollution in Berlin, Germany, leveraging data on PM10 from 2016 to 2019 provided by the Federal Environment Agency. We used data on this pollutant and years due to the availability of complete records provided by the closest station to the parliament (located in Berlin Center). Our analysis, show the impact of temperature to not change when controlling for air pollution, here included as a binary variable denoting 1 when exceeding the guidelines set by the WHO of 50 μg/m^3^ (Table S8).

### Conclusion

We believe that this study is an important first step in understanding the impact of environmental factors on language complexity leveraging large scale observational data on parliamentary speeches. Given the reality of climate change, the influence of extreme temperatures on language complexity, and more specifically on the quality of political discourse, raises significant concerns. The simplification of political discourse can be viewed as either beneficial or detrimental, depending on the context. Lower language complexity, for instance, can make political discourse easier to understand and help citizens become better informed about political choices.^29^ However, if a decrease in language complexity is indicative of reduced cognitive performance (as we propose in this study), these findings could suggest potential negative implications of climate change for the overall productivity of parliament members. Given the pivotal role that Members of Parliament play in democratic societies, a decrease in productivity could potentially lead to negative outcomes in crucial areas such as legislative decision-making, citizen representation, and budget planning. Consequently, the influence of extreme temperatures on politicians’ cognitive performance, even within the climate-controlled environment of a parliament, could have far-reaching negative implications for society as a whole.

## STAR★Methods

### Key resources table

**Table:** 

REAGENT or RESOURCE	SOURCE	IDENTIFIER
**Other**		

ERA5-Land Daily Aggregated - ECMWF Climate Reanalysis	Earth Engine Data Catalog	https://developers.google.com/earth-engine/datasets/catalog/ECMWF_ERA5_LAND_DAILY_AGGR
Data on daily PM10 in Berlin (Mitte)	German Environment Agency	https://www.umweltbundesamt.de/daten
German Parliamentary Data	Open Discourse Dataset	https://dataverse.harvard.edu/dataset.xhtml?persistentId=doi:10.7910/DVN/FIKIBO
US Congressional Record	Congressional Record for the 43rd-114th Congresses: Parsed Speeches and Phrase Counts	https://data.stanford.edu/congress_text
Other Countries’ Parliamentary Speeches	ParlSpeech V2	https://dataverse.harvard.edu/dataset.xhtml?persistentId=doi:10.7910/DVN/L4OAKN

**Software and algorithms**		

R Project for Statistical Computing	http://www.r-project.org/	
R package: tidyverse	https://CRAN.R-project.org/package=tidyverse	
R package: Quanteda	https://cran.r-project.org/web/packages/quanteda/index.html	
R package: STM	https://cran.r-project.org/web/packages/stm/index.html	

**Deposited data and code**		

Dataset and code for the replication of the main analysis	OSF	https://osf.io/jkqxz/; https://doi.org/10.17605/OSF.IO/JKQXZ

### Resource availability

#### Lead contact

Further information and requests for resources and reagents should be directed to and will be fulfilled by the lead contact, Risto Conte Keivabu (contekeivabu@demogr.mpg.de).

#### Materials availability

This study did not generate new unique reagents.

#### Data and code availability

The dataset and code to replicate the main analysis is deposited on OSF (https://osf.io) and is publicly available as of the date of publication (https://osf.io/jkqxz/).

### Method details

#### Parliamentary data and text analysis

The data for our dependent variables is based on parliamentary speeches from eight countries. The US congressional speeches and German parliamentary speeches are compiled in two separate datasets.^56^^,^^65^ Parliamentary speeches from the remaining countries (Austria, Denmark, Netherlands, New Zealand, Spain, and the UK) have been collected via the ParlSpeech dataset.^66^ In Table S2 we describe the research period, the number of legislators, and the number of speeches per country. We excluded from our sample the speeches made by parliamentary chairs and dropped all speeches that are shorter than 25 words in order to filter out further interjections and short questions from the plenary audience which could potentially influence our dependent variables.

To measure language complexity, we rely on different computational text analysis approaches. The main measure for language complexity in this study is the Flesch-Kincaid score, which is based on the Flesch score.^67^ The Flesch score is one of the most famous and widely used methods to assess the comprehensibility of texts and speeches (see for examples^28^^,^^38^). The Flesch score yields a score which lies typically in the range between 0 and 100, with 100 indicating texts that are very easy to read and 0 indicating texts that are very difficult to read (we report results for the main analysis with this measure in Figure S5). The formula to calculate the Flesch Score is given in Equation 1:

206.835−1.015×(totalwords/totalsentences)−84.6×(totalsyllables/totalwords)

The specific numbers in the Flesch Reading Ease formula, such as 206.835, 1.015, and 84.6, are constants that Rudolf Flesch derived empirically in the 1940s. Flesch developed the formula based on research into the factors that influence readability, focusing on sentence length (as a proxy for syntactic complexity) and word length (as a proxy for semantic complexity). The formula was designed to provide a quantitative measure of text difficulty that correlates with readability assessments made by human readers. Here's a brief overview of how these constants were determined: 206.835: This constant is a scaling factor intended to adjust the score to a 100-point scale, where higher values indicate easier readability. It was derived to set a baseline for the easiest readable texts under Flesch's original criteria. 1.015: This constant represents the weight given to the average sentence length (the number of words divided by the number of sentences). It reflects the impact of sentence complexity on the overall readability of a text. The specific value was determined through empirical testing to balance the influence of sentence length on the readability score. 84.6: This constant represents the weight given to the average word length in terms of syllables (the total number of syllables divided by the total number of words). It quantifies the impact of word complexity on readability. Like the other constants, this value was empirically determined to account for how word difficulty affects readability. Flesch's selection of these constants was based on his analysis of various texts, including their sentence and word lengths, and how these factors influenced readability for readers of different ages and educational levels. The constants were chosen to best fit the data available at the time, aiming to create a formula that could reliably predict the readability of English texts.

However, the Flesch score is typically converted to the Flesch-Kincaid score, which indicates the years of education generally required to understand a given text. The Flesch-Kincaid score is calculated as follows:

0.39×(totalwords/totalsentences)+11.8×(totalsyllables/totalwords)−15.59

The Flesch-Kincaid score is inverted with higher numbers indicating more complex sentences and lower numbers indicating fewer complex sentences. Both scores assess the “comprehensibility” (or complexity) of a text while considering the number of syllables relative to the number of words found in documents. The Flesch-Kincaid Grade Level formula is a readability test designed to indicate the U.S. school grade level required to understand a piece of text. It builds upon the work of Rudolf Flesch and was further developed by J. Peter Kincaid and his team under a contract with the U.S. Navy in the 1970s. The formula is specifically aimed at calculating the grade level, making it particularly useful in educational settings for assessing the suitability of texts for students. The formula outputs a number that corresponds to a U.S. grade level. For example, a score of 8.0 suggests that the text is suitable for an eighth-grade student, or around 13-14 years old. The constants in the formula serve specific roles: 0.39: This constant adjusts for the average sentence length (number of words divided by the number of sentences), contributing to the formula's measure of syntactic complexity. 11.8: This constant adjusts for the word complexity (number of syllables divided by the number of words), reflecting the formula's semantic complexity aspect. -15.59: These constant offsets the formula to align the grade level scale with the readability of typical English texts.

There are recent discussions about the appropriateness of the Flesch-Kincaid score to measure language complexity in political speeches. Critics point out that the Flesch-Kincaid score offers low construct validity because it largely relies on syntactic features of text (the structures of sentences) rather than semantic features.^68^ Furthermore, scores such as the Flesch-Kincaid have not been developed specifically for spoken language, but rather constitute measures of “reading ease”, i.e. reflecting the readability of written text.

However, the same authors also report correlations between complexity judgments of readers and Flesch-Kincaid scores.^68^ In addition, research from political science also shows that Flesch-Kincaid scores are good predictors of what humans understand as complex or difficult to understand. For instance, a study^69^ used short text snippets from US political speeches and asked crowd-coders to decide which of two text snippets is easier to understand. In a majority of these decisions, Flesch-Kincaid scores predicted correctly the snippet which humans perceived as easier. Similar results have been found in non-English language context. An experiment preregistration,^30^ replicated the above mentioned study^69^ using German-language text from German and Austrian party manifestos. Their findings show that the Flesch Reading Ease score performs “particularly well in explaining the perceived easiness of text snippets”.^30^

Lastly, in this study we are less concerned about language complexity per se. Instead, we perceive language complexity as a proxy for cognitive abilities of individual politicians measured over time.^41^ As stated in the introduction, we believe that extreme temperatures should affect the cognitive abilities of politicians which should make them less capable of forming long sentences including long words. We believe that for this purpose, Flesch-Kincaid scores constitute appropriate measures. Also, to further provide robustness to our measure we replicated our results using the Rix score that is based on two factors: the number of sentences and the number of long words (words with more than 6 letters) in a text.^70^ The main results show to replicate also using this indicator (Figure S5).

#### Meteorological data

We use as our main source of meteorological data information provided by the ERA-5 Land dataset. The ERA-5 Land dataset is gridded, provided by the Copernicus Data Store and it combines several sources of environmental data with sophisticated climate models to provide spatially and temporally comprehensive information.^71^ We collected daily data on precipitation, wind speed (u- and v- components), dew point temperature and mean temperature based on the grid located at the center of each of the cities hosting the parliaments. To do so, we accessed the data using the ERA-5 Land daily average values using Google Earth Engine providing data from January 2^nd^ 1950 to the present at resolution of about 11km. For Germany, we collect information for Bonn and Berlin as the location of the parliament changed after the reunification of East and West Germany in 1990 and the parliament was relocated from Bonn to Berlin on the 19th of April 1999. Considering the control variables, we computed relative humidity based on the daily mean temperature and dew point temperature for which we calculated the saturation vapor pressure based on the formula: E(T)=6.11×10(7.5×T/(237.7+T)) where T refers to temperature or dew point temperature. Followingly, we computed relative humidity dividing the saturation vapor pressure at the dewpoint temperature from the saturation vapor pressure at the air temperature and multiplied by 100. For wind speed, we retrieved both the u component (wind speed in the horizontal direction) and v component (wind speed in the vertical direction) and calculated wind speed as the square root of the squared u and v components.

### Quantification and statistical analysis

In this article we capture a plausibly causal effect of temperature on language complexity using an estimation strategy that has been applied in similar studies.^41^^,^^42^ More precisely, the estimation strategy is exposed in Equation 1:

(Equation 1) $$Y_{\text{ipct}}=\sum_{j}\beta \text{TEM}P_{\text{ct}}^{j}+X_{\text{tc}}\beta_{\text{ct}}+\delta_{\text{mc}}+\upsilon_{d}+\mu_{p}+\varepsilon_{\text{ipct}}$$

In the equation, Y_ipct_ denotes the outcome, Flesh-Kincaid score, measured for speech *i* of politician *p*, in city *c* at date *t.* The temperature exposure is captured by TEMP composed by the categories *j* < 0°C; 0°C to 3°C; 3°C to 6°C; 6°C to 9°C; 9°C to 12°C; 12°C to 18°C (comfort zone); 18 to 21°C; 21 to 24°C; 24 to 27°C; >27°C measured at city *c* and date *t.* As explained in the results section, we used politician level fixed effects, *μp* to isolate the effect of temperature variations on the speeches of individual politicians. The month-by-city fixed effects ***δ***_mc_ are used to account for seasonal patterns and the day of week fixed effects *υ*_*d*_ are used to rule out that our results are biased by possible weekly differences in speech quality. Also, we added a vector X for our control variables wind speed, relative humidity and precipitation. As in similar previous studies,^43^ we cluster standard errors at the month-by-city level, as this better captures the level of our treatment and allows us to account for potential correlation of regression errors that could arise within the same city across different months and account for underestimation of the variability in our estimates that could result from weather patterns or local events affecting multiple observations within the same city during the same month. Additionally, we tested robustness to alternative clustering at the politician level. Results with such alternative clustering strategies show to be consistent (Table S3).

For our marginal effects analysis for Germany, we use the same approach of our main analysis but add an interaction between our temperature measures and the gender and age categories. The estimation strategy is shown in Equation 2:

(Equation 2) $$Y_{\text{ipct}}=\sum_{j}\beta \text{TEM}P_{\text{ct}}^{j}\times \text{DEM}O_{p}+X_{\text{tc}}\beta_{\text{ct}}+\delta_{\text{mc}}+\upsilon_{d}+\mu_{p}+\varepsilon_{\text{ipct}}$$

Here, the temperature categories are interacted with the demographic variables DEMO that for Figure 2 represent the age categories and for Figure 3 is the gender of the politician *p.* As written in the results section, the age categories are four and based on quartiles of the age distribution of politicians in our sample that we described in the results section. Here, we cluster standard errors at the politician level as done in previous studies,^13^^,^^42^ due to limited numbers of clusters at the city level (we have observations on only Bonn first and only Berlin from 1999) and the problems raised by using month as a cluster due to treatment allocations in one month possibly similar to adjacent months. Nevertheless, we acknowledge the limitations of such approach that could possibly determine Type 1 errors due to spatial autocorrelation and reduce standard errors. The remaining of the model resembles Equation 1 as we added month-by-city FE, day of the week FE and politician FE. Nevertheless, due to the milder climate of Germany, our temperature categories are only 9: < 0°C; 0°C to 3°C; 3°C to 6°C; 6°C to 9°C; 9 to 12°C;12°C to 18°C (comfort zone);18 to 21°C; 21 to 24°C; >24°C.

Our results are robust to a set of sensitivity analysis. Firstly, as stated in the results sections, we used different sets of model specifications (Table S3). More precisely, we tested the introduction of a linear and cubic time trend constructed using a continuous value for the week of the speech, a continuous month-by-city FE, a less restrictive model specification where we used year-by-city FE. The results show to hold in all models, but we observe changes in the effect size and significance of the results that is smaller in a more conservative modelling approach compared to a less restrictive model. Such results are expected as the variation left in a more restrictive model is lower. Nevertheless, despite these analyses, we acknowledge that we are not able to fully capture week-of-study specific time trends that could still partly bias our results. Secondly, we used additional operationalizations of language complexity. Instead of the Flesch-Kincaid score, as mentioned previously, we used the Flesch and Rix score (Figure S6). Also, we used the temperature on the three days before and after the speech to test for lagged effects. In Figure S7, we observe an effect of hot days only on the day of the speech and not on days before or after, which corroborates our findings. Finally, to investigate whether changes in language complexity are not driven by a change in the topics discussed, we replicated the German analysis while controlling for speech topic. To measure topics in parliamentary debates, we ran a structural topic model.^72^ Topic modeling, a subset of machine learning and natural language processing (NLP), provides a way of distilling large volumes of text into smaller topic groups. This method operates under the assumption that documents are mixtures of topics, where a topic is defined by a probability distribution over words. Thus, it identifies clusters of words that frequently appear together across the corpus and interprets these clusters as indicative of different "topics." To do so, it relies on an iterative process, which relies on two key distributions: the probability of topics given a document (document-topic distribution) and the probability of words given a topic (topic-word distribution). At the start, words are randomly assigned to topics, but as the algorithm iterates, it refines these assignments based on the context provided by the document-topic and topic-word distributions. The goal is to maximize the likelihood that the observed words in the documents are generated by these distributions. Through this iterative refinement, words that frequently co-occur across the corpus naturally cluster into topics, while documents that share similar words strengthen their associations with these topics.

For our specific application, we did not predetermine the number of topics (k), which is a common requirement in many topic modeling applications. Instead, we adopted the approach by Lee and Mimno (2017) which employs spectral initialization techniques.^72^ This method stands out by using spectral decomposition on the document-feature matrix to infer the latent topic structure without prior specification of the number of topics. This approach circumvents the often subjective and iterative process of selecting an appropriate k value, thereby enhancing the objectivity and reproducibility of the topic modeling process. Upon applying this method, we identified 107 distinct topics within the parliamentary debate texts. This reveals the wide range of subjects covered in these debates, underscoring the complexity of legislative discourse. After the topic model was applied, we needed to relate the topics back to individual speeches to understand the main focus of each speech. To accomplish this, we used the highest θ value for each document, which represents the probability of each topic given the document. Assigning topics based on the highest θ value ensures that each speech is associated with the topic it most strongly relates to. Replicating the analysis including the categorical topic variable, we observe a slightly smaller effect size compared to the model without such control (Table S9). Nevertheless, the coefficient remains negative and statistically significant substantiating our main results.
