# Supplementary material for: The effect of temperature on language complexity: Evidence from seven million parliamentary speeches
Source: iScience. 2024 Jun 13;27(6):110106. doi: 10.1016/j.isci.2024.110106 (PMC11270029; doi:10.1016/j.isci.2024.110106)
Supplement: Document S1. Figures S1–S7 and Tables S1–S9 [file mmc1.pdf]

iScience, Volume *27*

Supplemental information

The effect of temperature on language complexity: Evidence from seven million parliamentary speeches

Risto Conte Keivabu and Tobias Widmann

Table S1. Summary Statistics, related to main text

	Mean	Standard deviation
Flesch-Kincaid score	11.44	4.28
Flesch score	50.18	17.51
Average sentence length	20.79	9.48
Average word length	1.6	0.17
Mean temperature °C	12.31	7.99
Wind speed (m/s)	3.04	2.07
Relative humidity (%)	73.17	13.43
Precipitation (mm)	0.002	0.01
Total politicians	28,523	
Total speeches	7,425,184	

Note: reported are the average values and standard deviation.

Table S2. Observations by country, related to STAR methods

Country	Research Period	Number of Legislators	Number of Speeches
Germany	1950 to 2019	3,680	404,561
USA	1950 to 2017	18,087	3,370,321
UK	1989 to 2019	2,150	1,617,330
Austria	1996 to 2018	840	88,314
Netherlands	1996 to 2018	1,190	847,780
New Zealand	1989 to 2019	447	546,853
Denmark	1996 to 2018	740	450,463
Spain	1996 to 2018	1,389	99,562
Total		28,523	7,425,184

Note: we report the period for which we have observations in each country, the number of legislators and the total number of speeches included in the analysis.

Table S3. Different model specifications, related to STAR methods

	(1) Flesch-Kincaid	(2) Flesch-Kincaid	(3) Flesch-Kincaid	(4) Flesch-Kincaid	(5) Flesch-Kincaid	(6) Flesch-Kincaid
< 0°C	0.001	-0.020	0.053	-0.002	-0.002	0.001
	(0.029)	(0.030)	(0.032)	(0.028)	(0.028)	(0.015)
0°C to 3°C	0.003	-0.001	0.030	-0.000	0.001	0.003
	(0.026)	(0.025)	(0.023)	(0.025)	(0.026)	(0.012)
3°C to 6°C	0.018	0.014	0.028	0.016	0.015	0.018
	(0.023)	(0.024)	(0.023)	(0.023)	(0.023)	(0.011)
6°C to 9°C	0.005	0.005	0.007	0.005	0.005	0.005
	(0.022)	(0.023)	(0.023)	(0.022)	(0.022)	(0.010)
9 to 12°C	0.013	0.020	0.011	0.009	0.008	0.013
	(0.018)	(0.018)	(0.021)	(0.018)	(0.018)	(0.009)
18 to 21°C	-0.030*	-0.021	-0.024	-0.029*	-0.029*	-0.030*
	(0.014)	(0.013)	(0.016)	(0.013)	(0.013)	(0.009)
21 to 24°C	-0.015	-0.008	-0.028	-0.014	-0.015	-0.015
	(0.021)	(0.017)	(0.024)	(0.020)	(0.020)	(0.011)
24 to 27°C	-0.047*	-0.040*	-0.058*	-0.045*	-0.046*	-0.047*
	(0.021)	(0.017)	(0.024)	(0.020)	(0.021)	(0.013)
>27°C	-0.050*	-0.070*	-0.070*	-0.047*	-0.049*	-0.050*
	(0.023)	(0.023)	(0.030)	(0.023)	(0.023)	(0.017)
Wind Speed	-0.000	-0.001	0.000	-0.000	-0.000	-0.000
	(0.004)	(0.004)	(0.004)	(0.004)	(0.004)	(0.002)
Relative Humidity	-0.000	0.000	-0.001	-0.000	-0.000	-0.000
	(0.000)	(0.000)	(0.000)	(0.000)	(0.000)	(0.000)
Precipitation	0.668	0.208	1.443	0.638	0.656	0.668
	(0.847)	(0.853)	(0.874)	(0.833)	(0.834)	(0.461)
**Fixed Effects:**						
Month-by-city FE	YES	NO	NO	YES	NO	YES
Continuous Month-by-City FE	NO	YES	NO	NO	NO	NO
Year-by-city FE	NO	NO	YES	NO	NO	NO
Week-by-city FE	NO	NO	NO	NO	NO	NO
Cubic city specific time trend FE	NO	NO	NO	NO	YES	NO
Linear city specific time trend	NO	NO	NO	YES	NO	NO
Day of week FE	YES	YES	YES	YES	YES	YES
Individual FE	YES	YES	YES	YES	YES	YES
**Standard Errors:**						
Month-by-city	YES	YES	YES	YES	YES	NO
Politician	NO	NO	NO	NO	NO	YES
Observations	7,425,184	7,425,184	7,425,184	7,425,184	7,425,184	7,425,184

Note: Note: the table shows the results of exposure to the temperature ranges on the Flesch-Kincaid score with different model specifications. Standard errors in parenthesis. Significance level: * p<0.05.

Table S4. Summary Statistics only for Germany, related to main text

	Mean	Standard deviation
Flesch-Kincaid score	14.64	4.76
Flesch score	27.42	18.12
Average sentence length	20.95	10.59
Average word length	1.87	.15
Mean temperature °C	8.52	6.59
Wind speed (m/s)	2.73	1.33
Relative humidity (%)	77.2	10.66
Precipitation (mm)	14.64	4.76
Age	50.03	8.91
Female (proportion)	0.19	
Total politicians	3,680	
Total speeches	404,561	

Note: reported are the average values and standard deviations for the sample of Germany.

Table S5. Temperature, Flesch-Kincaid and interaction with age groups in Germany, related to Figure 2

Variable	Flesch-Kincaid	Standard Error
< 0°C	0.029	(0.077)
< 0°C * 2nd quartile of age	-0.019	(0.097)
< 0°C * 3rd quartile of age	-0.040	(0.096)
< 0°C * 4th quartile of age	0.008	(0.090)
0°C to 3°C	0.060	(0.074)
0°C to 3°C * 2nd quartile of age	-0.021	(0.093)
0°C to 3°C * 3rd quartile of age	-0.049	(0.090)
0°C to 3°C * 4th quartile of age	0.039	(0.085)
3°C to 6°C	0.081	(0.064)
3°C to 6°C * 2nd quartile of age	0.009	(0.080)
3°C to 6°C * 3rd quartile of age	-0.062	(0.079)
3°C to 6°C * 4th quartile of age	-0.048	(0.075)
6°C to 9°C	0.050	(0.068)
6°C to 9°C * 2nd quartile of age	0.087	(0.086)
6°C to 9°C * 3rd quartile of age	0.024	(0.085)
6°C to 9°C * 4th quartile of age	-0.064	(0.078)
9 to 12°C	-0.035	(0.062)
9 to 12°C * 2nd quartile of age	0.099	(0.089)
9 to 12°C * 3rd quartile of age	0.100	(0.089)
9 to 12°C * 4th quartile of age	0.031	(0.080)
18 to 21°C	0.008	(0.076)
18 to 21°C * 2nd quartile of age	0.009	(0.108)
18 to 21°C * 3rd quartile of age	0.058	(0.109)
18 to 21°C * 4th quartile of age	0.048	(0.101)
21 to 24°C	0.088	(0.110)
21 to 24°C * 2nd quartile of age	-0.110	(0.154)
21 to 24°C * 3rd quartile of age	-0.307*	(0.150)
21 to 24°C * 4th quartile of age	-0.317*	(0.132)
>24°C	-0.406*	(0.172)
>24°C * 2nd quartile of age	0.111	(0.279)
>24°C * 3rd quartile of age	0.280	(0.293)
>24°C * 4th quartile of age	-0.078	(0.249)
Observations		404,561

Note: the table shows the results of exposure to the temperature ranges on the Flesch-Kincaid score with an interaction with the age categories of the politicians in Germany. We added month-by-location, day of week and politician FE. Moreover, we control for precipitation, relative humidity and wind speed. Standard errors clustered at the politician level reported in parenthesis. Baseline category is the first quartile of the age distribution. Significance level: * $p<0.05$.

Table S6. Temperature, Flesch-Kincaid and interaction with gender in Germany, related to Figure 3

	(1)<br>Flesch-Kincaid
< 0°C	0.086
	(0.077)
< 0°C * Female	-0.108
	(0.080)
0°C to 3°C	0.113
	(0.079)
0°C to 3°C * Female	-0.114
	(0.095)
3°C to 6°C	0.106
	(0.075)
3°C to 6°C * Female	-0.054
	(0.060)
6°C to 9°C	0.123
	(0.068)
6°C to 9°C * Female	-0.147*
	(0.062)
9 to 12°C	0.053
	(0.037)
9 to 12°C * Female	-0.073
	(0.048)
18 to 21°C	0.039
	(0.088)
18 to 21°C * Female	-0.088
	(0.081)
21 to 24°C	-0.138
	(0.119)
21 to 24°C * Female	0.007
	(0.140)
>24°C	-0.346*
	(0.089)
>24°C * Female	0.029
	(0.210)
Observations	404,561

Note: the table shows the results of exposure to the temperature ranges on the Flesch-Kincaid score with an interaction with the gender categories of the politicians in Germany. We added month-by-location, day of week and politician FE. Moreover, we control for precipitation, relative humidity and wind speed. Standard errors clustered at the politician level and reported in parentheses. Baseline category is men. Significance level: * p<0.05.

Table S7. Temperature and Flesch-Kincaid over time, related to main text

	(1)<br>Full sample	(2)<br>U.S.	(3)<br>Germany
21 to 24°C	-0.062	-0.044	-0.103
	(0.051)	(0.028)	(0.221)
21 to 24°C * 1968-1985	0.062	0.058	0.101
	(0.050)	(0.033)	(0.291)
21 to 24°C * 1986-2003	0.084	0.047	-0.021
	(0.062)	(0.033)	(0.253)
21 to 24°C * 2004-2019	0.040	0.088*	-0.062
	(0.061)	(0.037)	(0.229)
24 to 27°C (>24°C)	-0.091*	-0.076*	-0.743*
	(0.038)	(0.031)	(0.249)
24 to 27°C (>24°C) *1968-1985	0.052	0.050	0.789*
	(0.031)	(0.036)	(0.356)
24 to 27°C (>24°C) *1986-2003	0.115	0.065	0.415
	(0.060)	(0.036)	(0.336)
24 to 27°C (>24°C) *2004-2019	0.066	0.119*	0.043
	(0.057)	(0.039)	(0.276)
>27°C	-0.113*	-0.091*	
	(0.031)	(0.032)	
>27°C * 1968-1985	0.089*	0.079	
	(0.037)	(0.043)	
>27°C * 1986-2003	0.121	0.099*	
	(0.068)	(0.038)	
>27°C * 2004-2019	0.069	0.112*	
	(0.071)	(0.042)	
Observations	7,416,890	3,370,659	396,267

Note: the table shows the results of exposure to the temperature ranges on the Flesch-Kincaid score with an interaction with the year categories 1950-1967;1968-1985;1986-2003;2004-2019. In column (1), we run the analysis with the full dataset and we added month-by-location, day of week and politician FE. Moreover, we control for precipitation, relative humidity and wind speed. Standard errors clustered at the month-by city level and reported in parentheses. The sample is slightly smaller compared to the main analysis due to a higher number of singleton observations. In column (2), we run the model for the U.S. sample comprising data from 1950 to 2017. In column (3), we run the analysis for Germany comprising data from 1950 to 2019. For the analysis in column (2) and (3) we cluster standard errors at the politician level. The analysis includes all the temperature bins described in Equation (1), but reported are only the coefficient for the hotter days. For Germany, the highest temperature bin is >24°C. Baseline category is 1950-1967. Significance level: * p<0.05.

Table S8. Temperature, Flesch-Kincaid and controls for PM10 Germany, related to main text

	(1) Flesch-Kincaid	(2) Flesch-Kincaid
< 0°C	0.266*	0.291*
	(0.095)	(0.097)
0°C to 3°C	0.104	0.118
	(0.080)	(0.083)
3°C to 6°C	0.260*	0.290*
	(0.070)	(0.072)
6°C to 9°C	0.098	0.121
	(0.070)	(0.072)
9 to 12°C	0.175*	0.178*
	(0.061)	(0.064)
18 to 21°C	-0.112	-0.079
	(0.065)	(0.067)
21 to 24°C	-0.257*	-0.251*
	(0.070)	(0.073)
>24°C	-0.308*	-0.319*
	(0.145)	(0.146)
Wind Speed	-0.006	-0.004
	(0.015)	(0.015)
Relative Humidity	0.004	0.004
	(0.002)	(0.002)
Precipitation	1.449	0.907
	(2.016)	(2.061)
PM10 > 50 μg/m3		0.189
		(0.152)
Observations	34,181	34,181

Note: the table shows the results of exposure to the temperature ranges on the Flesch-Kincaid score in Germany. We added month-by-location, day of week and politician FE. Moreover, we control for precipitation, relative humidity, wind speed. Standard errors clustered at the politician and reported in parentheses. In the second column we add controls for PM10 being above 50 μg/m3. Significance level: * $p<0.05$.

Table S9. Temperature, Flesch-Kincaid and controls for Topic of the speeches in Germany, related to STAR methods

	(1) Flesch-Kincaid	(2) Flesch-Kincaid
< 0°C	0.066	0.068
	(0.076)	(0.065)
0°C to 3°C	0.092	0.101
	(0.075)	(0.071)
3°C to 6°C	0.096	0.095
	(0.071)	(0.066)
6°C to 9°C	0.096	0.090
	(0.071)	(0.067)
9 to 12°C	0.039	0.020
	(0.039)	(0.040)
18 to 21°C	0.020	0.014
	(0.081)	(0.074)
21 to 24°C	-0.136	-0.122
	(0.131)	(0.118)
>24°C	-0.343*	-0.292*
	(0.110)	(0.115)
Controls for Topic	NO	YES
Observations	404,561	404,561

Note: the table shows the results of exposure to the temperature ranges on the Flesch-Kincaid score in Germany. We added month-by-location, day of week and politician FE. Moreover, we control for precipitation, relative humidity and wind speed. Standard errors clustered at the politician and reported in parentheses. In the second column we add controls for the topic of the speech. Topics are in total 107. Significance level: * p<0.05.

Figures

Figure S1. Mean temperature over time by cities, related to main text

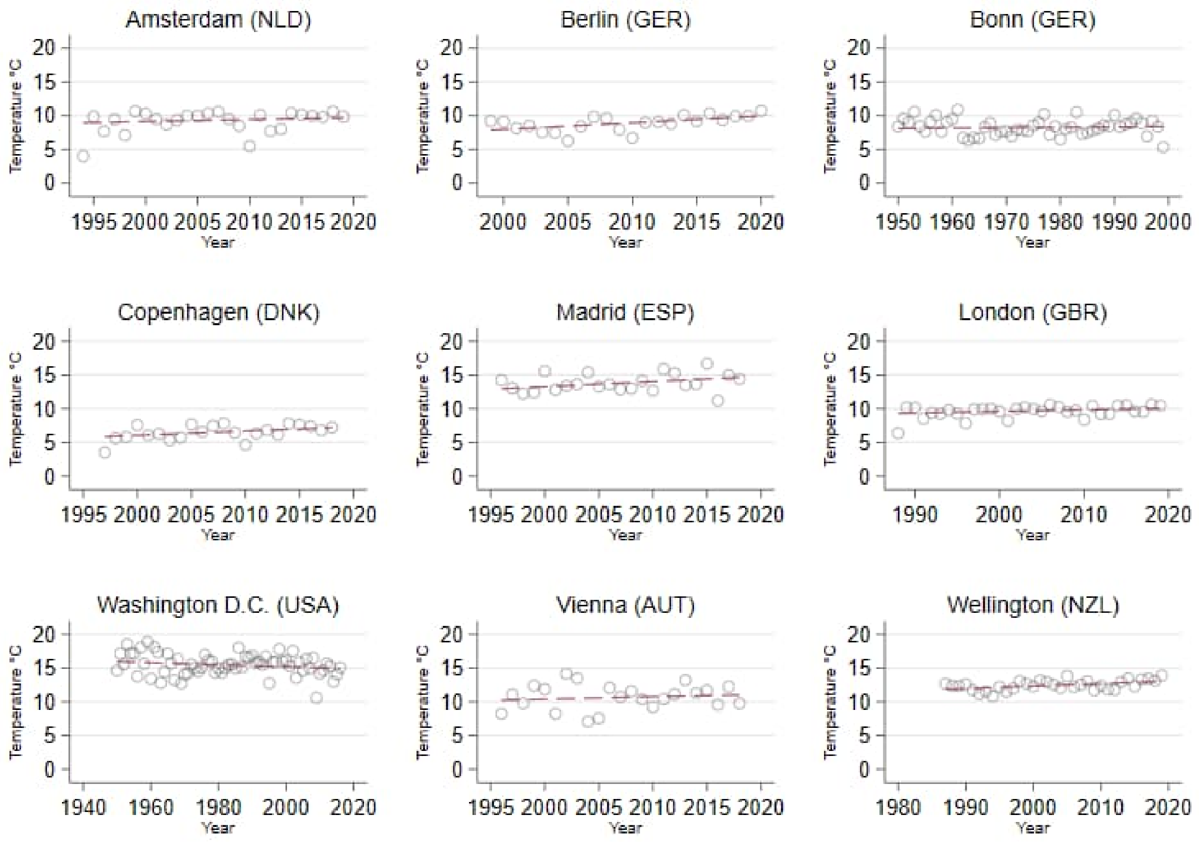

Note: In the figure we expose the trend in mean temperature by cities during the period of analysis.

Figure S2. Flesch-Kincaid score over time by cities, related to main text

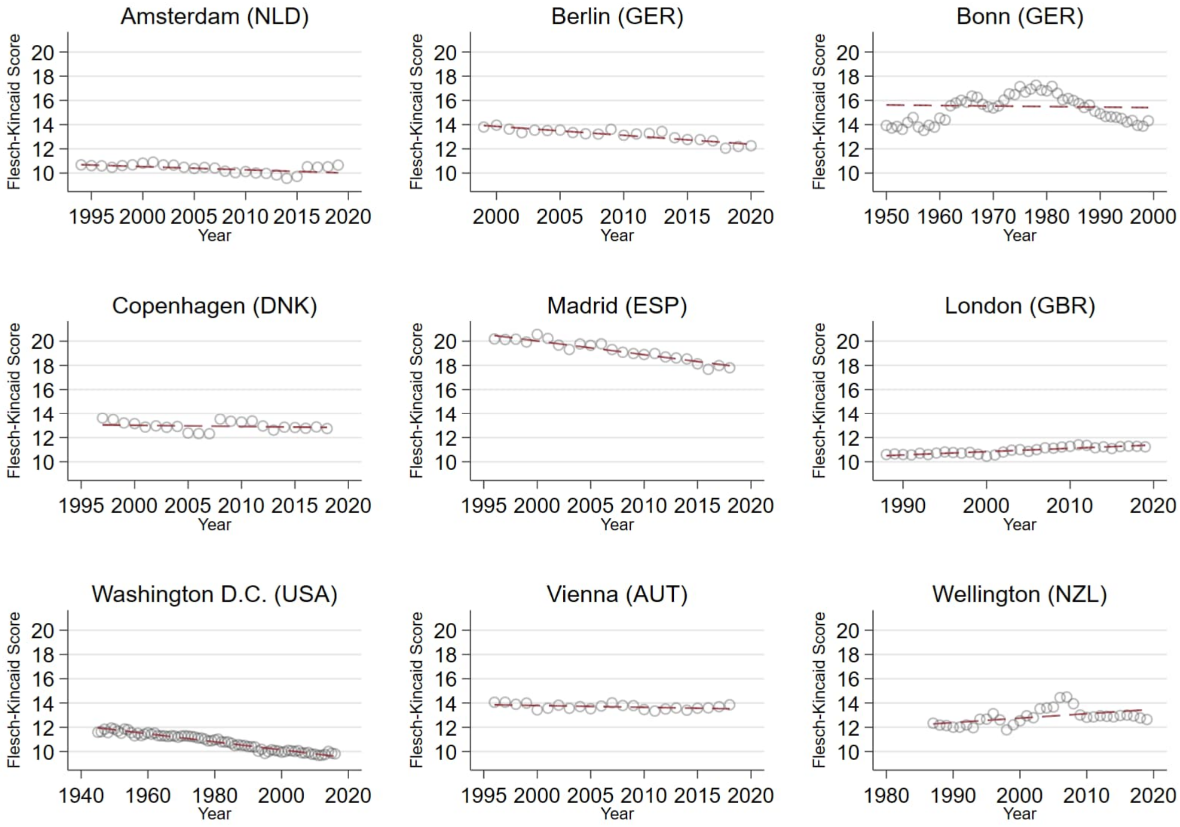

Note: In the figure we expose the trend in Flesch-Kincaid score by cities.

Figure S3. Exposure to each of the temperature categories in the estimation sample, related to main text

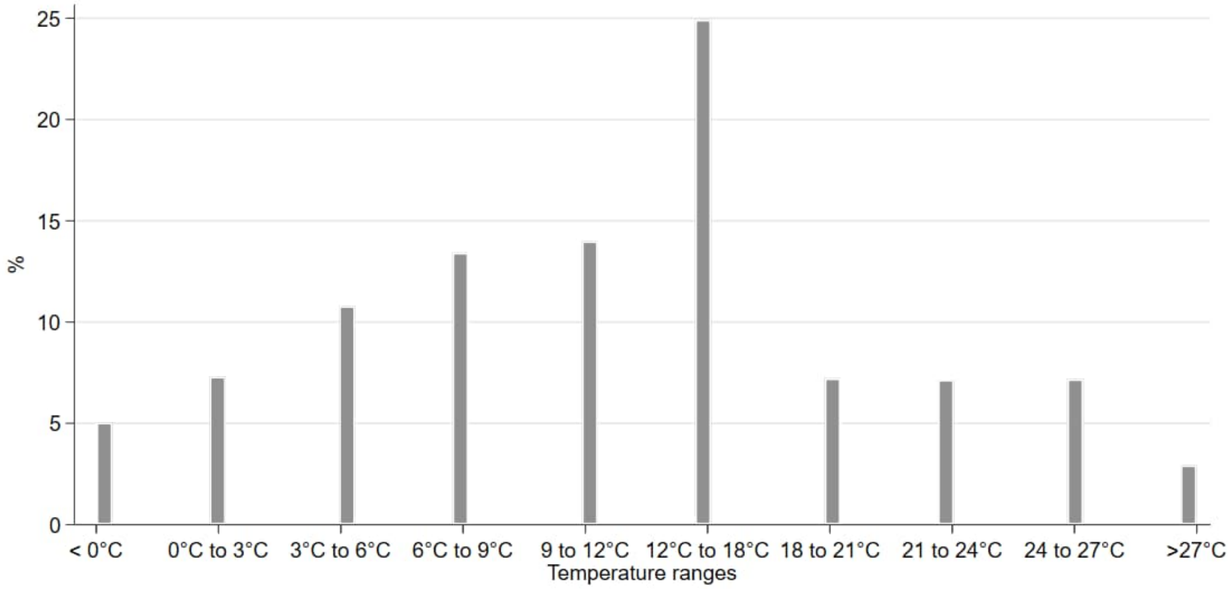

Note: displayed are the percentages of exposure to each of the temperature bins in the estimation sample.

Figure S4. Exposure to each of the temperature categories in the sample for Germany, related to main text

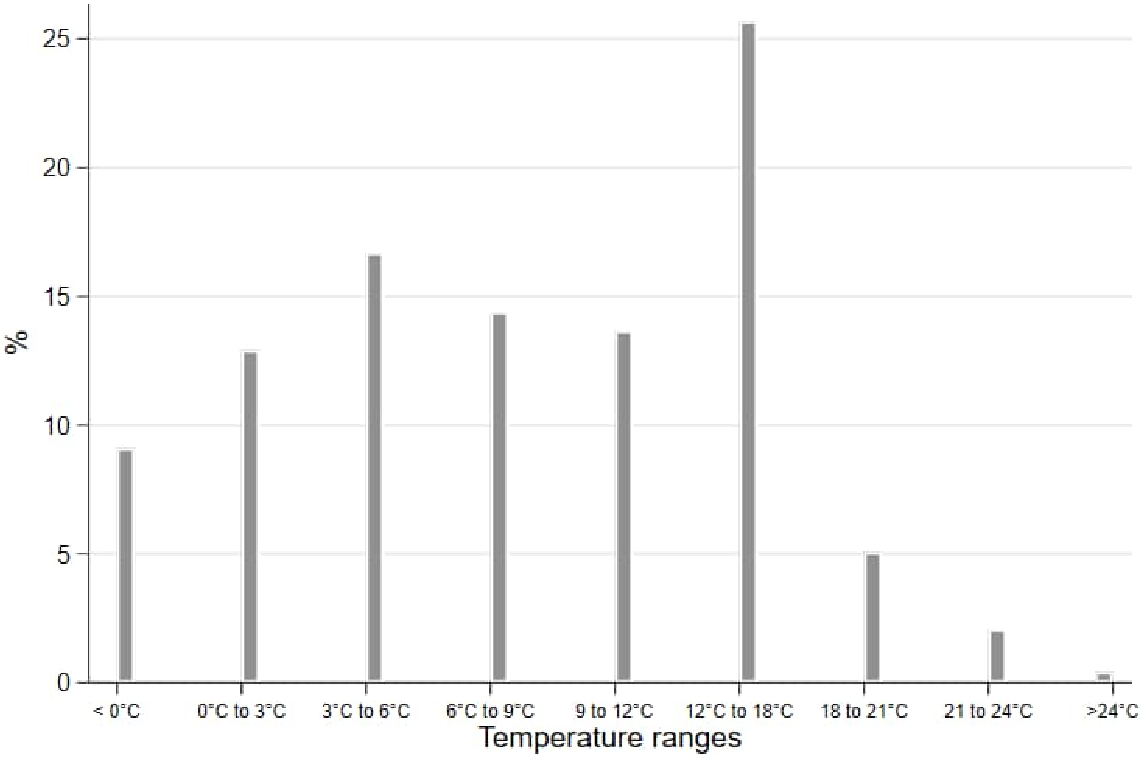

Note: displayed are the percentages of exposure to each of the temperature bins in the German sample.

Figure S5. Sentence length, word length and average temperature, related to main text

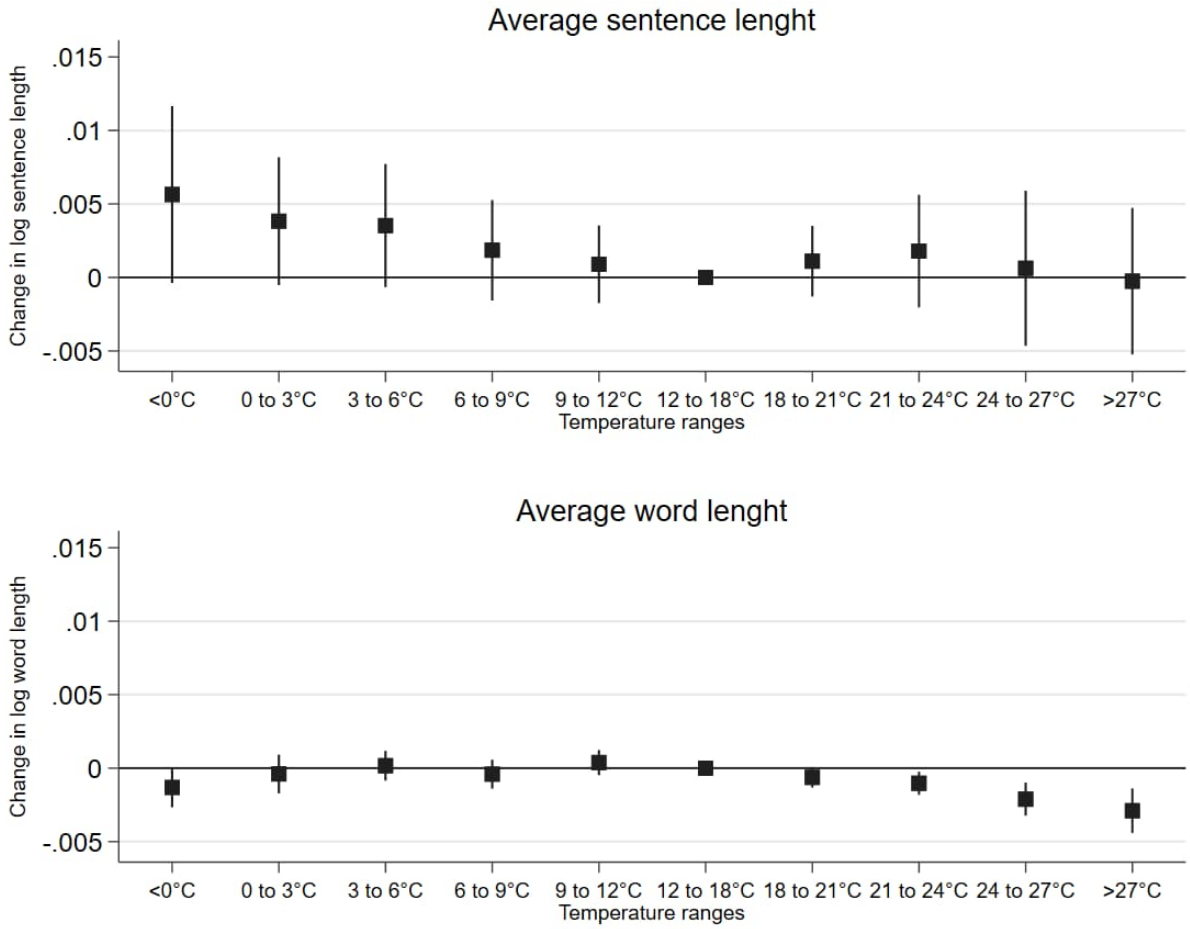

Note: the figure shows the results of exposure to the temperature ranges on log sentence length and log word length. We added month-by-location, day of week and politician FE. We control for precipitation, relative humidity and wind speed. Standard errors clustered at the month-by-location level. 95% confidence intervals.

Figure S6. Temperature exposure and change in Flesch score and Rix score for all countries, related to STAR methods

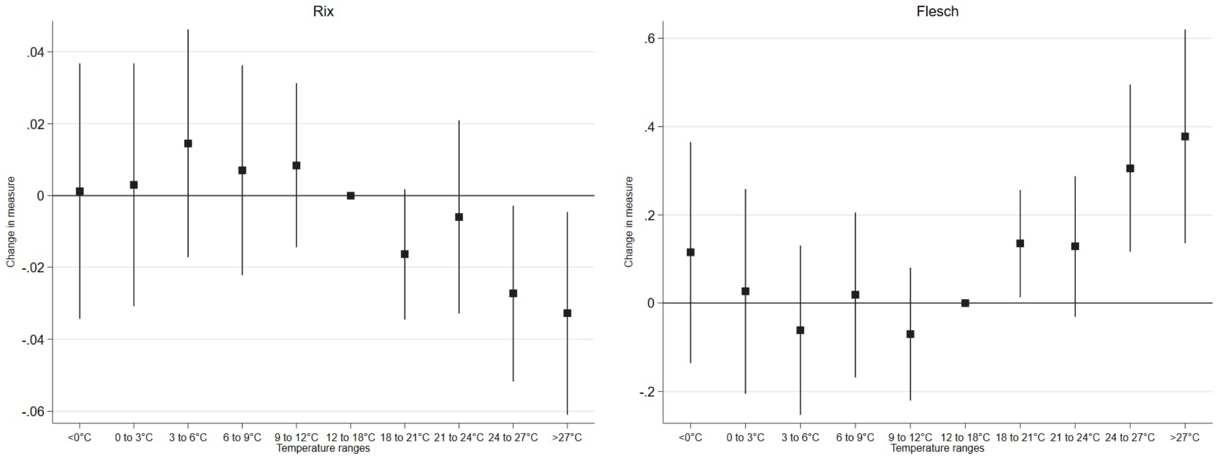

Note: the figure shows the results of exposure to the temperature ranges on the Flesch score that has an inverted scale compared to the Flesch-Kincaid indexes. We added month-by-location, day of week and politician FE. we control for precipitation, relative humidity and wind speed. Standard errors clustered at the month-by-location level. 95% confidence intervals.

Figure S7. Timing of exposure to high temperatures and Flesch-Kincaid score, related to STAR methods

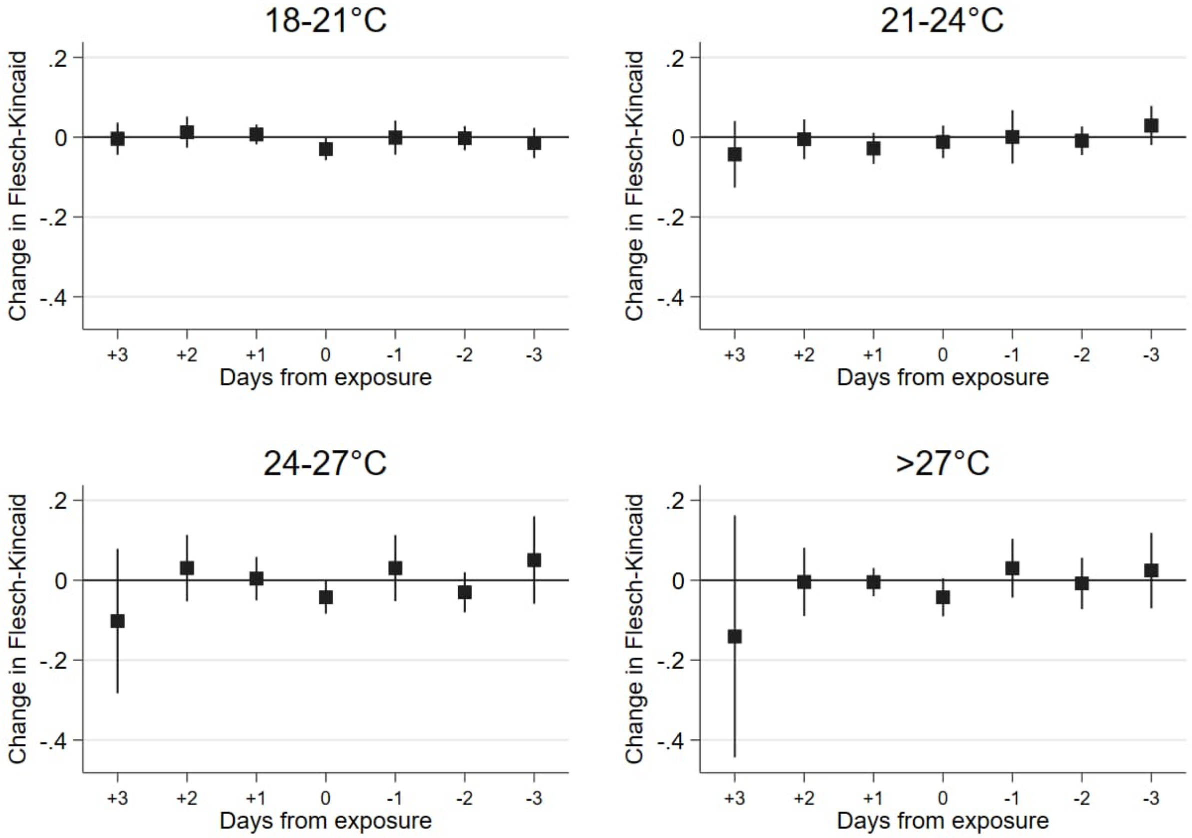

Note: the figure shows the results of exposure to the temperature categories above 18°C on the day of the speech and with exposure 3 days before and after the day of the speech on the Flesch-Kincaid score. The other temperature categories are included in the model but not reported. We added month-by-location, day of week and politician FE. We control for precipitation, relative humidity and wind speed. Standard errors clustered at the month-by-location level. 95% confidence intervals.
